# Effective management of hypertension with dihydropyridine calcium channel blocker-based combination therapy in patients at high cardiovascular risk

**DOI:** 10.1111/j.1742-1241.2008.01713.x

**Published:** 2008-05-01

**Authors:** H Haller

**Affiliations:** Department of Medicine, Division of Nephrology, Hannover Medical School Hannover, Germany

## Abstract

The increasing prevalence of hypertension, owing to modern lifestyles and the increasing elderly population, is contributing to the global burden of cardiovascular (CV) disease. Although effective antihypertensive therapies are available, blood pressure (BP) is generally poorly controlled. In addition, the full benefits of antihypertensive therapy can only be realised when target BP is achieved. International guidelines and clinical trial evidence support the use of combination therapy to manage hypertension. In high-risk patients, such as those with coronary artery disease, diabetes and renal dysfunction, BP targets are lower and there is a need for intensive management with combination therapy to control BP and provide additional CV risk reduction benefits.

Combinations of antihypertensive agents with different but complementary modes of action improve BP control and may also provide vascular-protective effects. Calcium channel blockers (CCBs) have been shown to be effective in combination with a range of antihypertensive drugs and in different patient populations. As part of a first-line combination strategy, CCBs can provide CV benefits beyond BP control, even in patients at increased CV risk. Benefits include protection against end-organ damage and serious CV events. Indeed, in major intervention trials, these benefits have already been clearly demonstrated. Ongoing studies will provide further data to support the clinical benefits of combination therapy as a first-line treatment approach. Implementation of this approach in clinical practice, together with adherence to global hypertension management guidelines will help ensure patients achieve and sustain BP targets, and reduce the risk of CV events.

**Review Criteria**

A literature search was conducted to identify recent randomised studies assessing CCB-based combination therapy strategies.

**Message for the Clinic**

Despite guideline recommendations, combination therapy is an underused strategy for the treatment of hypertension. Calcium channel blocker (CCB)-based combination strategies are effective and well tolerated when used with other classes of antihypertensive drugs, and should be considered a first-line option in hypertensive patients, particularly in those at high cardiovascular risk.

## Introduction

Hypertension is one of the most important modifiable causes of premature death worldwide, and is estimated to cause 7.1 million premature deaths. Approximately one billion people worldwide have hypertension and the prevalence is predicted to increase dramatically in the next few years (1,2).

Hypertension is a major risk factor for both cardiovascular (CV) and cerebrovascular morbidity and mortality (1), contributing to approximately 50% of all CV events (3). The relationship between blood pressure (BP) and CV risk is continuous – for every 20 mmHg increase in systolic blood pressure (SBP) or 10 mmHg increase in diastolic blood pressure (DBP), the risk of cardiovascular disease (CVD) doubles (4).

Patients with hypertension are also more likely to have associated CV risk factors (5); for example, approximately 50% of patients with hypertension have hypercholesterolaemia and 20–40% have hyperglycaemia. The presence of multiple risk factors increases the risk of CV events associated with hypertension. The most common risk factors for CVD include advanced age (> 55 years for men and > 65 years for women), smoking, dyslipidaemia, family history of premature CVD, abdominal obesity, abnormal C-reactive protein levels and clinical conditions such as diabetes and renal disease (6). As a result, current treatment guidelines emphasise the importance of risk stratification to determine BP targets and appropriate antihypertensive treatment regimens (6). For example, in patients with diabetes or other additional risk factors, the BP targets are lower: SBP < 130 mmHg and DBP < 80 mmHg, vs. SBP < 140 mmHg and DBP < 90 mmHg in patients with no additional risk factors (2,6,7).

The ultimate goal of hypertension management is to reduce CV morbidity and mortality by preventing end-organ damage (6–8). Numerous intervention studies have shown that BP control is associated with significant reductions in CV morbidity and mortality. Even modest reductions in SBP or DBP for short periods of time substantially improve CV outcomes, particularly in high-risk patients (9). For example, antihypertensive therapy is associated with a 35–40% reduction in stroke, a 20–25% reduction in myocardial infarction, a > 50% reduction in heart failure and reductions in CVD-related death rates (6,10). In addition to appropriate management of additional risk factors and associated clinical conditions, early, intensive and effective BP control is required in the prevention and management of CVD (6,8).

## Blood pressure control and achievement of guideline goals

Although effective therapies exist, current BP control is still below the ‘Healthy People 2010’ goal of 50%: only 34% of patients with hypertension have adequately controlled BP, 59% have treated but uncontrolled BP and 30% are unaware of their condition (7). Poorly controlled BP, particularly SBP, is associated with increased CV morbidity and mortality, and end-organ damage (11). Despite the availability of effective antihypertensive treatments, adequate BP control is often not achieved, highlighting the need for greater efforts in the management of hypertension.

Hypertension guidelines have traditionally recommended stepwise regimens to lower BP in patients with hypertension, beginning with lifestyle modification (e.g. weight reduction, increased physical activity, dietary changes, smoking cessation and moderation of alcohol consumption), and adding pharmacological intervention when lifestyle changes are insufficient (2,6,7,12). Immediate initiation of antihypertensive therapy, together with lifestyle changes is recommended in individuals at high or very high risk; whereas, for those at low or moderate risk, the effects of lifestyle changes should be monitored for several weeks before initiation of antihypertensive treatment (6). Guidelines also recommend that antihypertensive therapy should be started gradually to achieve target BP values progressively over several weeks.

Data from outcome studies show that several classes of drugs, including angiotensin-converting enzyme (ACE) inhibitors, angiotensin-receptor blockers (ARBs), beta-blockers, calcium channel blockers (CCBs) and thiazide-type diuretics, effectively lower BP and reduce the complications of hypertension (10,13–16). However, the recent ESH/ESC guidelines recommend that the presence of additional conditions, such as diabetes or coronary artery disease (CAD), or possible contraindications should be considered when selecting the initial antihypertensive agent (6).

In clinical practice, hypertension management varies greatly and many factors contribute to inadequate BP control, the most important include: patient non-compliance; acceptance of inadequate BP control by clinicians and reluctance to titrate the dose, switch to another drug or add another drug; and the fact that it is difficult to achieve adequate BP control with monotherapy in most patients, even when the dose is optimised (17). Response rates with any class of antihypertensive administered as monotherapy range from 30% to 60%; however, no monotherapy has been shown to achieve target BP in more than 20–30% of the overall hypertensive population (18,19). By contrast, combining two complementary antihypertensive agents has been shown to improve the response rate to 75–90% (17), and the results of the Anglo-Scandinavian Cardiac Outcomes Trial (ASCOT) showed that about nine of 10 patients required two or more antihypertensive agents to reduce BP to < 140/90 mmHg (20). Therefore, most patients, particularly those at high CV risk, will require combination therapy with two or more antihypertensive medications to achieve controlled BP, and recent guidelines recommend that two-drug combination therapy be considered a first-line alternative to monotherapy (2,6,7).

Compared with high-dose monotherapy, combination therapy is associated with fewer adverse effects. Guidelines advocate combination therapy with once daily treatment regimens that provide 24-h efficacy. The advantages of combination therapy include improved adherence to therapy (21) and minimisation of BP variability. In addition, combining two antihypertensive agents with different mechanisms of action may provide greater protection against major CV events and the development of end-organ damage (6). The challenge remains to translate the evidence and recommendations outlined in the current hypertension management guidelines into clinical practice, as combination therapy remains underused, especially in high-risk patients (22).

## Combination therapy – the evidence

**Figure 1:**
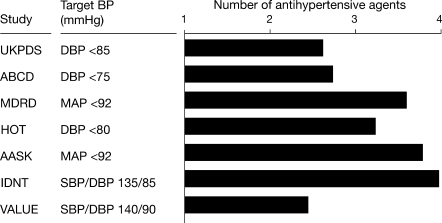
Two to four antihypertensive agents are required to achieve effective BP control to target levels. UKPDS, United Kingdom Prospective Diabetes Study (23); ABCD, Appropriate Blood Pressure Control in Diabetes (27); MDRD, Modification of Diet in Renal Disease study (28); HOT, Hypertension Optimal Treatment study (24); AASK, African American Study of Kidney Disease and Hypertension (29); IDNT, Irbesartan Diabetic Nephropathy Trial (26); VALUE, Valsartan Antihypertensive Long-term use Evaluation Trial (25); BP, blood pressure; DBP, diastolic blood pressure; MAP, mean arterial pressure; SBP, systolic blood pressure

Combination therapy was traditionally reserved as a third- or fourth-line approach in hypertension management (7); however, several major intervention trials in various high-risk patient populations have shown that an average of two to four antihypertensive agents are required to achieve effective BP control to target levels (Figure 1) (23–29). A significant level of support for combination therapy is also provided by monotherapy studies in which additional or background antihypertensive therapy was required to effectively lower BP (Table 1) (13,14,16,24–26,30–35).

**Table 1:** The high use of combination therapy in major monotherapy trials

Study	Duration (years)	Number of patients	Main drug	Comparator drugs	Patients receiving combination therapy (%)
ACTION (34)	4.9	7665	Nifedipine	Placebo	80% beta-blocker; 20% ACE inhibitor; 2% ARB;12% diuretic; 3% other
ALLHAT (13)	4.9	33,357	Amlodipine	Lisinopril; chlorthalidone;doxazocin	71% of the amlodipine group; 80% of the lisinopril group68% of the chlorthalidone group
CAMELOT (32)	2	1997	Amlodipine	Enalapril; placebo	31% diuretic; 76% beta-blocker; 9% ACE inhibitor;8% CCB; 2% ARB
EUROPA (31)	4.2	12,218	Perindopril	Placebo	62% beta-blocker; 32% CCB; 9% diuretic
HOPE (16)	5	9297	Ramipril	Placebo	47% CCB; 40% beta-blocker; 15% diuretic
HOT (24)	3.8	18,790	Felodipine	No comparator	41% ACE inhibitor; 28% beta-blocker 22% diuretic
IDNT (26)	2.6 (meanfollow-up)	1715	Irbesartan	Amlodipine; placebo	Both treatment arms received on average 3 non-studydrugs*
INVEST (33)	2.7 (meanfollow-up)	22,576	Verapamil	Atenolol trandolapril†; HCTZ†	67% of the verapamil group and 69% of theatenolol group received 2 or 3 strategy drugs
LIFE (14)	4	9193	Losartan	Atenolol	66% of the losartan group; 62% atenolol group
RENAAL (30)	3.4	1513	Losartan	Placebo	77.9% CCB (60.7% DHP CCB); 83.8% diuretic; 40.2%alpha-blockers; 34.1% beta-blockers; 18% centrally actingagents
Syst-Eur (35)	2.0	4695	Nitrendipine	Placebo enalapril‡;HCTZ‡	38% enalapril; 18% HCTZ
Syst-China (39)	3	1253	Nitrendipine	Placebo	19% captopril; 3% HCTZ; 3% other
VALUE (25)	4.2	15,245	Valsartan	Amlodipine	In the valsartan vs. amlodipine groups: 21 vs. 19% ACE inhibitor;24 vs. 18%α-blocker; 48 vs. 43% beta-blockers; 13 vs.15% diuretics; 4 vs. 4% diuretic combinations

* Non-study drugs to control BP included diuretics, beta-blockers, peripheral alpha blockers and central α_2_ antagonists.

† Add-on therapy administered to achieve BP goals.

‡ Add-on therapy to nitrendipine. ACE, angiotensin-converting enzyme; ARB, angiotensin-receptor blockers; CCB, calcium channel blocker; HCTZ, hydrochlorothiazide.

Guidelines recommend various two-drug combinations of different classes of antihypertensive agents based on data derived from controlled interventional trials, but advise that three or four drugs may be required depending on the patient's risk profile. Although older therapies such as diuretics and beta-blockers can effectively lower BP and are included as possible first-line combinations, they are associated with some disadvantages (6). For example, beta-blockers offer no benefit to elderly patients with uncomplicated hypertension. Furthermore, they may be associated with an increased risk of stroke (36) and impaired glucose and lipid metabolism (37). These studies recommend that beta-blockers should not be first choice for the treatment of uncomplicated hypertension. When diuretics are administered at higher doses or in combination with beta-blockers, they are associated with increased risks of new-onset diabetes (37). Evidence to support the use of diuretics as first-line treatment has also been questioned and these concerns are reflected in recent guidelines (6).

By contrast, many studies have shown that newer antihypertensive agents, such as CCBs, ARBs and ACE inhibitors, provide additional benefits by reducing the incidence of CV events in patients with hypertension (14,16,25,30). In addition, cases of new-onset diabetes are less common with newer antihypertensive agents than with older therapies such as diuretics and beta-blockers (37). Whether this is due to the deleterious effect of older agents on glucose metabolism or a positive effect of newer agents remains to be fully determined.

## CCBs in combination therapy – evidence supporting additional treatment benefits

### Combination studies

Calcium channel blockers are used extensively in clinical practice and data from several clinical studies show that CCBs effectively and safely lower BP and reduce long-term CV risk in a wide range of patient populations (24,32,35,38,39). It is of note that while most studies have investigated the efficacy and safety of dihydropyridine CCBs, there are some studies supporting the benefits of non-dihydropyridine CCBs (33,40,41). However, for the purpose of this review, data presented on CCBs are for dihydropyridine CCBs.

As CCBs have a different mode of action to commonly used inhibitors of the renin–angiotensin–aldosterone (RAAS) pathway (such as ACE inhibitors and ARBs), combination with these agents should provide synergistic or complementary effects, compared with using two agents that inhibit the same pathway. Indeed, in patients with newly diagnosed stage 1 or 2 hypertension or in patients with inadequate BP control after conventional low-dose monotherapy, low-dose combination therapy with CCBs and ARBs was found to provide better BP control than either high-dose monotherapy (p < 0.05 vs. either monotherapy) (42). Further evidence for the benefits of CCB/ARB combination therapy was provided by Kuschnir et al. (43), who showed that the combination of low-dose nifedipine gastrointestinal therapeutic system (GITS) with losartan was associated with improved BP control (greater and more consistent) than either monotherapy (p < 0.05) in patients with mild-to-moderate hypertension. Similarly, in hypertensive patients in the Nifedipine and Candesartan Combination (NICE-Combi) study, low-dose CCB/ARB combination therapy with nifedipine controlled-release (CR) and candesartan was shown to be more effective than up-titrated candesartan monotherapy for both BP control and renal protection, with significant reductions in urinary microalbumin excretion levels with combination therapy compared with either monotherapy (p < 0.05) (38). The Japanese Adalat CR and Valsartan Cost-Effectiveness Combination (ADVANCE-Combi) study was conducted to extend the findings of the NICE-Combi study and determine the optimal CCB (nifedipine CR vs. amlodipine) for combination therapy with valsartan in patients with essential hypertension. BP was significantly reduced in both treatment arms, but to a greater extent in patients receiving nifedipine CR and valsartan than in those receiving amlodipine and valsartan (p < 0.01) (44).

In the Systolic Evaluation of Lotrel Efficacy and Comparative Therapies study, CCB and ACE inhibitor combination therapy with amlodipine and benazepril was significantly more effective in reducing SBP and pulse pressure in patients with severe systolic hypertension than either monotherapy (p < 0.0001) (45). Significantly greater percentages of patients in the combination group achieved reductions in BP to guideline-recommended targets compared with either monotherapy (p < 0.0001) (17). These findings are supported by those of a similar study that investigated a CCB and ACE inhibitor combination in patients with hypertension who were inadequately controlled on monotherapy. The combination of manidipine and delapril was shown to be more effective in reducing BP than either drug alone. At the end of the treatment period, 73% of patients achieved controlled BP (46). Efficacy and safety data from the key studies comparing CCB combination therapy to monotherapy are presented in Table 2 (38,42,43).

**Table 2:** Comparison of efficacy and safety of CCB combination therapy vs. monotherapy in clinical trials

Study	Efficacy	Safety
NICE-Combi: nifedipine and candesartan low-dosecombination therapy vs. candesartan monotherapyin patients with essential hypertension (38)	BP reduction significantly greater in combinationarm (SBP 12.1 mmHg, DBP 8.7 mmHg) vs.monotherapy arm (SBP 4.1 mmHg,DBP 4.6 mmHg)	Decreased urinary microalbumin excretion incombination arm (p < 0.05) but not monotherapyarm
High-dose monotherapy vs. low-dose combinationtherapy of CCBs and ARBs (42)	In patients whose hypertension not controlled withmonotherapy, low-dose combination therapyachieved BP control in 61.6%, vs. 42.8% withhigh-dose CCBs and 40.5% with ARBs Combination therapy exhibited bettertrough-to-peak variability, hypertensive burdenand BP variability	Low-dose combination therapy better toleratedthan high-dose CCB monotherapy
Low-dose nifedipine GITS and losartan in patientswith mild-to-moderate hypertension (43)	DBP lower in patients receiving combinationtreatment vs. losartan alone DBP trough-to-peak ratio and smoothness indexhighest in combination group (70%)	Adverse events similar between treatment groups

ARB, angiotensin-receptor blockers; BP, blood pressure; CCB, calcium channel blocker; DBP, diastolic blood pressure; GITS, gastrointestinal therapeutic system; NICE-Combi, Nifedipine and Candesartan Combination; SBP, systolic blood pressure.

### Add-on studies

Data from outcome trials show that CCB therapy plus additional add-on treatment not only lowers BP but also improves patient outcomes. The Hypertension Optimal Treatment trial showed that intensive lowering of BP with CCB-based therapy (felodipine as baseline therapy with the addition of other antihypertensive agents according to a five-step regimen) was associated with a low rate of CV events (22). In the Systolic Hypertension in Europe (Syst-Eur) and China (Syst-China) studies, the dihydropyridine CCB nitrendipine, with the addition of a diuretic and an ACE inhibitor (enalapril in Syst-Eur and captopril in Syst-China) reduced the rate of CV complications in elderly patients with isolated systolic hypertension (35,39). The additional benefits of CCB-based therapy in the elderly patients were further supported by the Shanghai Trial Of Nifedipine in the Elderly, in which nifedipine GITS reduced the incidence of CV events, including stroke, in elderly individuals with hypertension (47).

Data from ASCOT showed that CCB-based treatment is more effective than a beta-blocker-based regimen for reducing mortality and CV events (20). ASCOT compared the combination of the CCB amlodipine plus the ACE inhibitor perindopril (added as required) with the beta-blocker atenolol plus the diuretic bendroflumethiazide (added as required) in a group of patients with hypertension and at least three other CV risk factors (20). Treatment with a CCB-based therapy reduced the risks of non-fatal myocardial infarction or fatal CHD (p = 0.046), fatal and non-fatal stroke (p = 0.0003), total CV events and procedures (p < 0.0001), all-cause mortality (p = 0.025) and diabetes (p < 0.0001) compared with the beta-blocker-based therapy (20). Thus, the CCB-based regimen prevented more major CV events and was associated with a reduced incidence of diabetes compared with the diuretic-based regimen. The results of ASCOT therefore support the benefits of combined antihypertensive therapy for lowering BP and significantly reducing the risk of CV events.

### Additional studies

In addition, there are several ongoing studies to determine the optimal antihypertensive combination therapy with the most favourable safety profile for lowering BP and protecting against CV events. The Combination Therapy of Hypertension to Prevent Cardiovascular Events study is a multicentre trial assessing CV outcomes in hypertensive patients treated with various drug combinations including ARBs, beta-blockers or diuretics in combination with a CCB (benidipine) (48). The Avoiding Cardiovascular Events Through Combination Therapy in Patients Living with Systolic Hypertension trial is the first randomised controlled trial to compare the effects of ACE inhibitor/diuretic and ACE inhibitor/CCB first-line combination therapies (benazepril/hydrochlorothiazide vs. benazepril/amlodipine) in hypertensive patients with additional CV risk factors, including renal disease and diabetes (49). Results from these trials should provide new evidence to select optimal combination therapies for hypertensive patients.

Several other large, randomised clinical studies have investigated the use of antihypertensive combination therapies in high-risk patient populations, including those with impaired renal function, diabetes or CAD, in whom BP is more difficult to control to target levels. These will be discussed in more detail in the following sections.

### Patients at risk of stroke

There is strong evidence to show that hypertension is probably the most important risk factor for stroke (50) – the risk of stroke increases linearly with increasing BP (51). In patients at risk of stroke, reducing BP has a significant benefit: a 5–6 mmHg reduction in BP has been shown to reduce the risk of stroke by 38% (52). The CCB-based antihypertensive strategies in particular have been shown to provide specific benefits in patients at risk of stroke (47). The combination of a CCB and an ACE inhibitor has been shown to significantly reduce the risk of stroke in elderly patients with isolated systolic hypertension, with relative risk reductions vs. placebo of 38% (p = 0.01) and 42% (p = 0.003) in Syst-Eur and Syst-China respectively (35,39). More recently, ASCOT demonstrated a greater reduction in the risk of non-fatal or fatal stroke with CCB plus additional ACE inhibitor therapy compared with a beta-blocker plus additional diuretic therapy (p = 0.0003) (20). In A Coronary Disease Trial Investigating Outcome with Nifedipine GITS (ACTION), the addition of the CCB nifedipine GITS to best practice therapy for CAD, including other antihypertensive agents, reduced the risk of debilitating stroke by 33% in hypertensive patients (p = 0.029) compared with hypertensive patients who did not receive additional CCB therapy (49).

## Benefits of CCB-based therapy in high-risk patient populations

The safety and efficacy of CCBs have also been demonstrated in several high-risk patient populations, such as those with impaired renal function, diabetes or CAD.

### CCB combination therapy and renal function

In hypertensive patients at high CV risk, renal dysfunction has been shown to be an important predictor of CV risk and to act as a prognostic marker of progression to CVD (53). Furthermore, BP is more difficult to control in patients with impaired renal function, particularly in those with comorbid diabetes. Importantly, tight BP control has been shown to slow the progression of renal failure (54).

ACTION examined the benefits of additional nifedipine GITS intervention in patients with stable angina and CAD who were receiving best practice therapy. Almost 40% of the patients in ACTION had evidence of renal dysfunction (34,55), and the results showed that, when administered in addition to best practice therapy for CAD, nifedipine GITS significantly reduced BP (p < 0.0001 compared with placebo) even in patients with renal dysfunction in whom it is more difficult to achieve BP control.

In the International Nifedipine GITS Study: Intervention as a Goal in Hypertension Treatment (INSIGHT), patients with mild-to-moderate hypertension received nifedipine GITS or the diuretic combination co-amilozide, with the addition of atenolol (or enalapril if atenolol was contraindicated) followed by addition of any other antihypertensive drug (other than CCBs or diuretics) if BP targets were not achieved. The study showed that, in hypertensive patients at high CV risk, renal function was better preserved with nifedipine GITS than with diuretics (p < 0.0001). The improved renal function with nifedipine GITS was indicated by better preserved creatinine clearance, which is a marker of renal function, and fewer patients treated with nifedipine GITS had progressive renal deterioration compared with those treated with co-amilozide (34,53).

Further evidence of the benefits of CCBs in patients with impaired renal function was provided by the Antihypertensive and Lipid-lowering Treatment to Prevent Heart Attack Trial, which included a *post hoc* analysis of the changes in estimated glomerular filtration rate with a CCB (amlodipine), a diuretic (chlorthalidone) and an ACE inhibitor (lisinopril). In this trial, the incidence of end-stage renal disease was similar for all three treatment arms, but estimated creatinine clearance was significantly better preserved with amlodipine than with chlorthalidone or lisinopril (13). Taken together, the results of these studies demonstrate that dihydropyridine CCBs confer prognostic benefits in terms of renal function.

### CCB combination therapy and diabetes

Individuals with hypertension have a ≥ 2-fold increased risk of developing diabetes (56). In addition, hypertension is twice as common in patients with diabetes compared with those without diabetes; it accounts for up to 75% of CV risk in this patient population, leading to substantial increases in morbidity and mortality (57).

Hypertension acts synergistically with diabetes in increasing the risks of macro- and microvascular diabetic complications (58,59). In patients with type 1 diabetes mellitus, hypertension is often the result of underlying nephropathy; whereas in those with type 2 diabetes mellitus, hypertension may be present as part of the metabolic syndrome (60).

Owing to the increased CV risk associated with diabetes, target BP is lower in patients with diabetes (130/80 mmHg) than in patients with primary hypertension (5,6). However, fewer than one-third of individuals with diabetes achieve BP targets, in part because of the inherent difficulty of controlling BP in these patients (59). Current guidelines recognise the importance of achieving target BP levels and recommend that all patients with diabetes and hypertension should be treated with a combination of several antihypertensive drugs (one of which should be an ARB or ACE inhibitor) (6,61).

Several studies have shown that CCB-based combination therapy can improve clinical outcomes in individuals with diabetes. In INSIGHT, nifedipine GITS reduced the risk of all-cause mortality in patients with diabetes compared with those without diabetes and reduced the frequency of new cases of diabetes compared with diuretic therapy (62). Further support for CCB-based combination therapy in patients with diabetes is provided by ACTION, the results of which showed that nifedipine GITS, in addition to best practice therapy for CAD, significantly reduced BP and the risk of CV events (34). The Reduction of Endpoint in NIDDM with the Angiotensin II receptor Antagonist Losartan study investigated the addition of losartan to current hypertensive medication in patients with diabetes and diabetic nephropathy. Most patients were taking dihydropyridine CCBs, and the addition of losartan led to significant renal-protective effects, with a risk reduction of 28% in end-stage renal disease (p = 0.002). This benefit was beyond that attributable to the BP-lowering effect alone (30,51).

### CCB combination therapy and CAD

In ACTION, more than 50% of patients had inadequately controlled BP at baseline, despite receiving best practice therapy at study entry (34). At baseline, 20% of patients were receiving ACE inhibitors; ≥ 10% were receiving diuretics; 2% were receiving ARBs and ≥ 80% were receiving beta-blockers. The addition of nifedipine GITS to best practice CAD therapy provided further benefit by increasing the proportion of patients who achieved BP targets: the percentage of patients with BP above target was reduced from 52% at baseline to 35% in the nifedipine GITS group and 47% in the placebo group. The effects of nifedipine GITS on BP lowering resulted in improved patient outcomes, which were mainly attributable to reductions in stroke or transient ischaemic attack and the need for coronary angiography or coronary interventions (34). The benefits of nifedipine GITS intervention were also evident in those patients with additional complications, including patients with underlying atrial fibrillation (63).

Further analysis of the ACTION results revealed even greater benefits in the subgroup of patients with inadequate BP control at baseline (64). There was a significant 13% reduction (p < 0.05) in the combined incidence of all-cause mortality, myocardial infarction, refractory angina, heart failure, debilitating stroke and peripheral revascularisation in patients receiving nifedipine GITS in addition to best practice therapy. In addition, a 38% reduction in new overt heart failure and a 33% reduction in the incidence of debilitating stroke were observed in patients treated with nifedipine GITS. Together, these data clearly indicate that long-acting CCBs are an effective and well-tolerated combination therapy choice in high-risk patients with CAD.

## Benefits of antihypertensive drug combinations with complementary modes of action

Hypertension is a multifactorial disease, so disruption of a single physiological pathway is often insufficient to control BP. Therefore, a combination of two drugs with different but complementary modes of action is often needed to achieve effective BP control. This is supported by hypertension guidelines, which highlight the need for drugs to be combined effectively and emphasise the benefits of drugs with different mechanisms of action on a multi-regulated variable such as BP (2,6,7).

Dihydropyridine CCBs and inhibitors of the RAAS pathway (namely, ACE inhibitors and ARBs) are widely used in patients with CVD. They have complementary mechanisms of action so, when used in combination, have synergistic effects on pathological changes in the vasculature and end organs, providing benefits in addition to BP control.

Calcium channel blockers primarily affect the cellular interactions of endothelial cells, smooth muscle cells, monocytes and thrombocytes, which have key roles in the early phases of atherosclerosis development. There is also evidence to show that CCBs affect the nitric oxide system in endothelial cells. Several studies have shown that endothelium-dependent relaxation, which is impaired in individuals with hypertension, can be restored by treatment with dihydropyridine CCBs (65–67). CCBs also have vascular-protective effects, which are evident during the later stages of atherosclerosis. The International Nifedipine Trial on Anti-atherosclerotic Therapy study demonstrated a 28% reduction in new atherosclerotic lesions in patients with mild CAD with nifedipine GITS (68). In patients with significant atherosclerosis, even greater reductions in lesions are observed with a CCB and statin combination, as shown in the Regression Growth Evaluation Statin Study (69). CCBs have also been shown to have beneficial effects on early carotid wall changes by reducing intima-media thickness (70–72), and can improve coronary endothelial function in patients with CAD (73). By contrast, ARBs and ACE inhibitors act on the RAAS hormone pathway to block signalling and promote relaxation of blood vessels, thereby controlling BP and providing beneficial effects on CV morbidity and mortality in high-risk patients and preserving renal function. In addition, it was recently shown that ARBs have a specific anti-inflammatory effect by reducing levels of inflammatory markers such as tumour necrosis factor-α, interleukin-6 and C-reactive protein (74). In addition to effective BP lowering, combination therapy with a CCB and an ARB would be expected to provide additional benefits through effects on both oxidative stress and microinflammation.

## Conclusions

Both guideline recommendations and clinical trial evidence support the use of combination therapy in managing hypertension, particularly in patients at increased risk of CV events and those for whom BP targets are lower because of the need for intensive management of additional risk factors, such as those with CAD, metabolic syndrome, diabetes or renal dysfunction. In a large proportion of patients, it is difficult to lower BP to target levels using antihypertensive monotherapy, so more intensive intervention, specifically combination antihypertensive therapy, is often required. Clinical studies have proved the efficacy of CCB-based combination strategies in a wide range of high-risk patient groups.

Combination therapy with antihypertensive agents that have different but complementary mechanisms of action not only avoids unnecessary drug interactions and adverse events, but also maximises the benefits of agents that have additional effects beyond BP lowering. For example, there is evidence to show that the combination of CCBs and ARBs provides end-organ protection through synergistic mechanisms. CCBs are effective with all other antihypertensive agents, and this flexibility makes them ideal as part of a first-line combination strategy to achieve target BP and provide additional CV benefits, without compromising safety in patients at increased CV risk.

In clinical practice, there is still much inconsistency with regards to stepwise treatment of patients and the decision to increase drug dose, switch therapy or add another drug. Combination therapy may still be considered a last resort in the treatment of hypertension and be rarely used as initial or first-line therapy. The treatment paradigm is now changing and more patients are being treated in line with guideline recommendations, which focus on overall CV risk and therefore recommend multiple-drug strategies early in the course of treatment.

Ongoing studies will provide further data to support the benefits of antihypertensive combination therapy on clinical outcomes when used as a first-line strategy. Increased efforts to use combination therapy much earlier in the course of treatment and increased adherence to global guidelines will ensure patients achieve and sustain BP control in addition to reducing the risk of CV events.
